# The biogeography of the yeti crabs (Kiwaidae) with notes on the phylogeny of the Chirostyloidea (Decapoda: Anomura)

**DOI:** 10.1098/rspb.2013.0718

**Published:** 2013-08-07

**Authors:** C. N. Roterman, J. T. Copley, K. T. Linse, P. A. Tyler, A. D. Rogers

**Affiliations:** 1 Department of Zoology, University of Oxford, South Parks Road, Oxford OX1 3PS, UK; 2 Ocean and Earth Science, University of Southampton, Waterfront Campus, Southampton SO14 3ZH, UK; 3 British Antarctic Survey, High Cross, Madingley Road, Cambridge CB3 0ET, UK

**Keywords:** Kiwaidae, Chirostyloidea, biogeography, phylogenetics, hydrothermal vents, yeti crab

## Abstract

The phylogeny of the superfamily Chirostyloidea (Decapoda: Anomura) has been poorly understood owing to limited taxon sampling and discordance between different genes. We present a nine-gene dataset across 15 chirostyloids, including all known yeti crabs (Kiwaidae), to improve the resolution of phylogenetic affinities within and between the different families, and to date key divergences using fossil calibrations. This study supports the monophyly of Chirostyloidea and, within this, a basal split between Eumunididae and a Kiwaidae–Chirostylidae clade. All three families originated in the Mid-Cretaceous, but extant kiwaids and most chirostylids radiated from the Eocene onwards. Within Kiwaidae, the basal split between the seep-endemic *Kiwa puravida* and a vent clade comprising *Kiwa hirsuta* and *Kiwa* spp. found on the East Scotia and Southwest Indian ridges is compatible with a hypothesized seep-to-vent evolutionary trajectory. A divergence date estimate of 13.4–25.9 Ma between the Pacific and non-Pacific lineages is consistent with Kiwaidae spreading into the Atlantic sector of the Southern Ocean via the newly opened Drake Passage. The recent radiation of Kiwaidae adds to the list of chemosynthetic fauna that appear to have diversified after the Palaeocene/Eocene Thermal Maximum, a period of possibly widespread anoxia/dysoxia in deep-sea basins.

## 1. Introduction

The taxon-rich Anomura, an infraorder of decapod crustaceans, has been subjected to major taxonomic revisions in recent years [1–3]. This is especially true for squat lobsters (anomurans with a proportionally elongated abdomen only partially folded under the thorax), which used to be grouped together with porcelain crabs in the superfamily Galatheoidea [4]. Morphological re-examinations and molecular phylogenetics have revealed that the squat lobster form probably evolved independently at least twice from hermit crab-like forms within Anomura [5,6]. One clade, the Galatheoidea [1], now only comprises the squat lobster families Galatheidae, Munididae and Munidopsidae and the porcelain crabs, Porcellanidae, while the other clade comprises the superfamilies of the freshwater squat lobster Aegloidea, the marine squat lobster Chirostyloidea and the hairy stone crabs (Lomisoidea) [5]. These two groups form larger clades with Paguroidea (hermit crabs), a superfamily now shown to be polyphyletic [5].

The recently described marine squat lobster superfamily Chirostyloidea consists of three families: Chirostylidae, Eumunididae and the chemosynthetic-associated Kiwaidae (yeti crabs). Chirostylidae are divided into five genera (*Chirostylus*, *Gastroptychus*, *Uroptychus*, *Uroptychodes* and *Hapaloptyx*), while Eumunididae contains *Eumunida* and *Pseudomunida*. Kiwaidae are solely represented by the genus *Kiwa* [3]. The phylogenetic relationship among chirostyloid families and their genera is still unclear; analyses of three rRNA ribosomal genes and morphological characters by Schnabel *et al*. [6] indicated that *Eumunida* was nested in a clade comprising *Uroptychus*, *Uroptychodes*, *Gastroptychus* and *Chirostylus*, with *Kiwa* and *Pseudomunida* falling out basally, thus challenging the monophyly of Eumunididae. Despite these results, morphological evidence and recent work using the cytochrome oxidase subunit 1 gene (COI) still supports the monophyly of Eumunididae [4]. Comprehensive morphological examination of the sternal plastron in species of *Gastroptychus* [7] suggests two groups: one as *Gastroptychus sensu stricto*, and a second group, superficially similar to *Gastroptychus* s.s, which may have a closer affinity to some species of *Uroptychus* [6].

**Figure 1.**
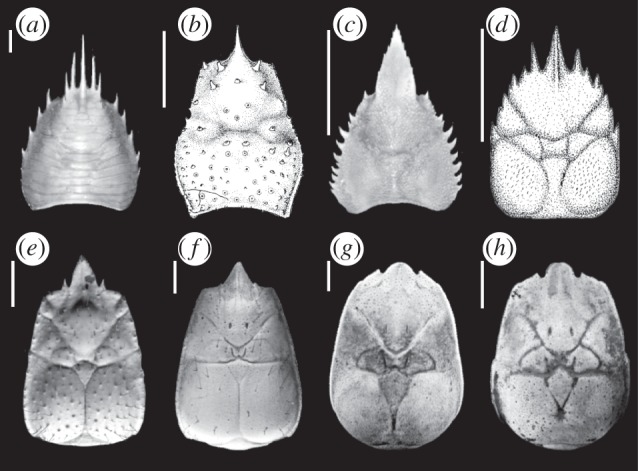
Modified photographs and illustrations of extant and extinct chirostyloid carapaces: (*a*) *Eumunida australis* (Eumunididae) modified from Schnabel & Ahyong [3], (*b*) *Gastroptychus iaspis* (Chirostylidae) from Baba & Haig [8], (*c*) *Uroptychus naso* (Chirostylidae) from Poore & Andreakis [9], (*d*) fossil chirostyloid *Pristinaspina gelasina* from Schweitzer & Feldmann [10], (*e*) *K. puravida* (Kiwaidae) from Thurber *et al*. [11], (*f*) *K. hirsuta* (Kiwaidae) from Macpherson *et al*. [2], (*g*) *Kiwa* n. sp*.* ESR, original photograph, (*h*) *Kiwa* SWIR, original photograph. Scale bars, 1 cm.

Using five nuclear protein-coding genes across Anomura, Tsang *et al*. [5] found support for a eumunidid–kiwaid clade as sister to Chirostylidae. This study used three species (*Kiwa hirsuta*, *Eumunida funambulus* and *Uroptychodes grandirostris*) to represent the chirostyloid families. A eumunidid–kiwaid clade is supported by the shared presence of supraocular spines (figure 1), an epipod bearing maxilliped 1 and a distally annulated flagellum on the exopod [3,4].

**Figure 2.**
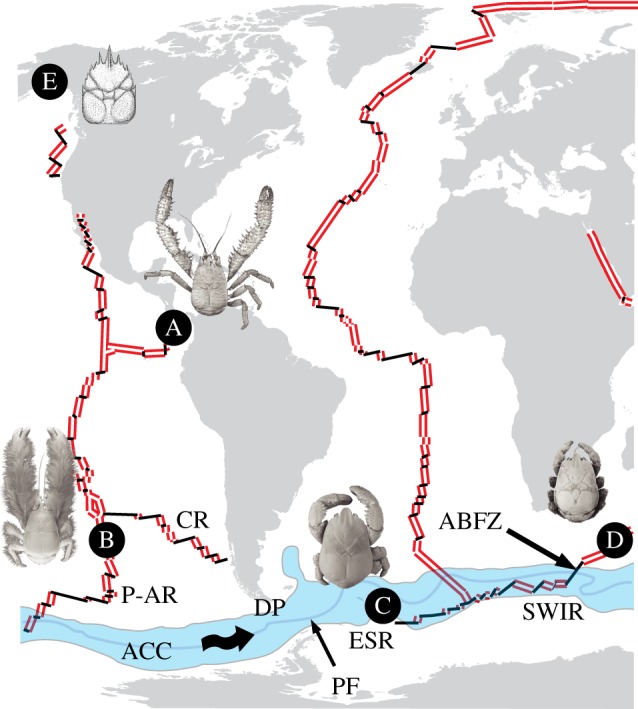
Map showing locations of kiwaids, each with representative image, (A, *K. puravida*; B, *K. hirsuta*; C, *Kiwa* n. sp*.* ESR; D, *Kiwa* SWIR), as well as the location of the fossil *Pristinaspina gelasina* (E) in relation to mid-ocean ridges (MORs) and the ACC. Double lines denote actively spreading MOR segments; single black lines represent intervening faults and fracture zones. Land shapes and ridge positions are modified from the NASA Digital Tectonic Activity Map [12]. Spreading ridge abbreviations are as follows: P-AR, Pacific–Antarctic Ridge; CR, Chile Rise; ESR, East Scotia Ridge; SWIR, South West Indian Ridge; ABFZ, Andrew Bain Fracture Zone. Shaded area labelled ACC, Antarctic Circumpolar Current as defined by the Subantarctic Front to the north and the Southern ACC front to the south. PF, Polar Front. Wavy arrows illustrate direction of the ACC. DP denotes the Drake Passage. Photographs of *K. puravida* and *K. hirsuta*, courtesy of Shane Ahyong from Thurber *et al*. [11] and Macpherson *et al*. [2], respectively. (Online version in colour.)

Kiwaidae, found exclusively in deep-sea chemosynthetic ecosystems, incorporates four species of the genus *Kiwa*, of which two are recently described [2,11]. *Kiwa hirsuta*, the type species for the genus and family, was found adjacent to hydrothermal vents on the Pacific–Antarctic Ridge in 2005 (figure 2). Based on its elongated, setae-covered chelae and a distinctly regionalized carapace, among other characters, a new family was described [2]. The profusion of apparently chemosynthetic filamentous bacteria found among the setae led Macpherson *et al*. [2] to speculate that kiwaids may be partly reliant on these bacteria as a source of nutrition, which was later confirmed [13]. In 2006, a second species, *Kiwa puravida*, was discovered at methane cold seeps on the Pacific continental slope off Costa Rica. Isotope analysis revealed the main diet to be epibiotic bacteria growing on carapace setae, which are scraped off by a specialized third maxilliped ‘comb’. *Kiwa puravida* is similar in form to *K. hirsuta*, and molecular characterization based on COI and rRNA 18S sequences confirms their close affinity [11].

A third undescribed species of *Kiwa* was discovered in 2010 in the Atlantic sector of the Southern Ocean at vents on the East Scotia Ridge (ESR) [14]. Compared with the first two species, it has proportionally much shorter chelae, with the majority of the bacteria-growing setae concentrated on the ventral carapace. rRNA sequences confirmed that *Kiwa* n. sp. ESR is closely related to *K. hirsuta* (6.45% divergence for 16S) [14]. In December 2011, a further *Kiwa* species, morphologically similar to *Kiwa* n. sp. ESR, was discovered at the Dragon hydrothermal vent field on the Southwest Indian Ridge (SWIR) [15].

The nature and timing of chirostyloid evolution is still unresolved; the fossil record of Chirostyloidea is poor, in contrast to Galatheoidea, for which there are fossils dating back to the Early Jurassic [4]. Currently, only one fossil has been attributed to Chirostyloidea: *Pristinaspina gelasina*, a fossil recovered from Cenomanian to Maastrichtian deposits in Alaska [10]. The animal was buried in a muddy continental slope environment at present-day latitude (approx. 60° N), which is quite different from either the chemosynthetic environments of extant Kiwaidae or the deep-water coral and sponge habitats with which many Chirostylidae and Eumunididae are believed to be associated [7]. Originally thought to be a chirostylid, the distinctive carapace regionalization characteristic of kiwaids, along with a broad medially carinate rostrum and supraorbital spines, indicate that this animal is possibly a stem-lineage kiwaid [4] (figure 1). It has been suggested that the northeast Pacific location of the fossil, along with the present-day location of *K. hirsuta* and *K. puravida*, reflect an East Pacific origin for the family [4].

This study aims to resolve phylogenetic uncertainties in the Chirostyloidea, and in particular Kiwaidae, by analysing a concatenated nine-gene ribosomal and protein-coding DNA sequence dataset in order to: (i) confirm the monophyly of Chirostyloidea and test the monophyly of Kiwaidae–Eumunididae; (ii) investigate polyphyly within Chirostylidae; (iii) reveal the internal phylogeny of Kiwaidae; (iv) date the key divergences in Chirostyloidea; and (v) relate divergences in Kiwaidae to past tectonic and oceanographic events.

## 2. Material and methods

### (a) Taxon sample set

Species of *Kiwa*, *Eumunida*, *Uroptychus*, *Gastroptychus*, *Uroptychodes* and *Chirostylus* have been included in this study. Only the monotypic *Pseudomunida* and *Hapaloptyx* genera in Eumunididae and Chirostylidae, respectively, are omitted, owing to tissue rarity. Non-chirostyloid anomurans have been chosen based on the most recent molecular phylogenies of Anomura [5,6] in order to provide fossil calibrations for estimating divergences within Chirostyloidea.

In total, 23 species were included in this study, featuring 15 chirostyloids, six other anomurans and two brachyurans (true crabs) as outgroups. Of the chirostyloids, nine species are chirostylids, two are eumunidids and four are kiwaids (see the electronic supplementary material, table S1 for information on tissue provenance and GenBank accession nos). New sequences have been deposited in GenBank under the nos KF051278–KF051401.

### (b) Molecular methods

Total genomic DNA was extracted from pereopods, pleopods or antennae using either Qiagen DNeasy Blood and Tissue Kit following the manufacturer's instructions or, in cases where tissue quantities were very small, a CTAB DNA extraction protocol [16]. Nine gene sequence regions were selected in this study: fragments of the ribosomal rRNA genes 16S (approx. 500 bp), 18S (approx. 1900 bp) and 28S (approx. 300 bp), as well as approximately 500 bp fragments of each of the protein-coding genes COI, arginine kinase (AK), enolase, glyceraldehyde 3-phosphate dehydrogenase (GAPDH), sodium potassium ATPase α subunit (NaK) and phosphoenolpyruvate carboxykinase (PEPCK). Of these genes, two are mitochondrial (16S and COI). Primers for these genes, including 15 newly designed, are listed in the electronic supplementary material, table S2.

PCR reactions were performed in 12 µl volumes, containing 0.8 µl of each primer (forward and reverse) at a concentration of 4 pmol µl^−1^, 8 µl of Qiagen HotStarTaq Master Mix, 2 µl of DNA template (approx. 10–50 ng µl^−1^) and 0.4 µl of double-distilled water. All PCR reactions were performed on a Bio-Rad C1000 Thermal Cycler.

General amplification conditions were initial HotStarTaq denaturation at 95°C for 15 min, followed by 35 cycles of 94°C for 1 min, 50°C for 90 s, 72°C for 1 min and a final extension of 72°C for 10 min. PCR product was visualized on 1 per cent agarose gel using ethidium bromide and then purified either using the QIAquick gel purification kits or Diffinity RapidTips. Sequencing reactions were performed in 10 µl volumes, containing 2.5 µl cleaned PCR product, 2 µl H_2_O, 2.5 µl of 0.8 pmol µl^−1^ primer, 2.5 µl 6X Buffer and 0.5 µl BigDye. The following sequencing reaction protocol was used: initial denaturation at 96°C for 1 min, followed by 25 cycles of 96°C for 10 s, 50°C for 5 s, 60°C for 4 min and a final cool down to 4°C.

Sequences were resolved using an Applied Biosystems 3100 Genetic Analyzer. Consensus sequences were generated from forward and reverse strands using Geneious Pro v. 5.4.6. [17].

Protein-coding genes (COI, NaK, enolase, AK, GAPDH and PEPCK) were aligned using the geneious alignment tool in Geneious Pro v. 5.4.6, and ribosomal genes (16S, 28S and 18S) were aligned using MAFFT 6 [18] and then adjusted by eye. Difficult-to-align variable regions in the rRNA sequences were excised using Gblocks [19]. The remaining gaps in the alignments were considered to be potentially informative and were coded for, using the FastGaps program [20]. The resulting gap-coding blocks were pasted to the ends of each rRNA sequence in the concatenated alignment to yield the final sequence dataset.

The final concatenated alignment is as follows: 16S (518 bp), 18S (1681 bp), 28S (232 bp), COI (585 bp), NaK (582 bp), enolase (339 bp), AK (600 bp), GAPDH (522 bp) and PEPCK (501 bp), resulting in a concatenated total alignment of 5560 bp, which is available online at TreeBASE (http://purl.org/phylo/treebase/phylows/study/TB2:S14238).

### (c) Partitioning and substitution model choice

To avoid multiple phylogenetic analyses on a shortlist of possible partition strategies, PartitionFinder [21] was used to evaluate the best partition scheme and accompanying substitution models according to the Akaike information criterion (see the electronic supplementary material, table S3).

### (d) Phylogenetic analyses

Two different methods for determining phylogenies were performed in this study: maximum likelihood (ML) and Bayesian inference (BI). ML analyses were performed using GARLI v. 2.0 [22], with two replicate runs, each with 200 bootstrap pseudo-replicates to determine node support. BI was performed using MrBayes v. 3.2 [23]. Metropolis-coupled Monte Carlo Markov chains (MCMC) were run for 10 million generations in two simultaneous runs, each with four differently heated chains. Convergence of the analyses was validated by the standard deviation of split frequencies and by monitoring of the likelihood values over time using Tracer v. 1.5 [24]. Topologies were sampled every 1000 generations and the first 2500 trees (25%) were discarded as ‘burn in’.

### (e) Topology hypothesis testing

Given the uncertainty regarding the affinity of Kiwaidae, Eumunididae and Chirostylidae within Chirostyloidea, three alternative *a priori* topological hypotheses were tested using the assessment of the marginal model likelihoods with the stepping-stone method in MrBayes v. 3.2 [25]. The topology hypotheses are as follows: a Kiwaidae–Eumunididae clade, a Kiwaidae–Chirostylidae clade and a Eumunididae–Chirostylidae clade. For each topology constraint, two simultaneous analyses were performed for 2.5 million generations, with default settings.

### (f) Divergence estimation using fossil calibration

Bayesian estimation of divergence times was performed with Beast v. 1.7.4 [26] for the entire concatenated dataset. Substitution models and clock models were unlinked across the partitions. Tuning parameters for the MCMC operators were set to auto-optimize and successive runs were tuned accordingly. Each MCMC chain commenced from a starter tree based on the topology of the phylogenetic trees created in §2*e* and run for 50 million generations. Two independent runs were performed; each sampled every 1000 generations, and 10 per cent of samples were removed as burn-in. Runs were combined using LogCombiner v. 1.7.4. Effective sample size values were greater than 200 for all parameters.

### (g) Fossil calibrations

*Pristinaspina gelasina* was not included as a fossil calibration point for kiwaid divergence, given the lack of any definitive proto-chirostyloid fossils for comparison and its shared features with Eumunididae. However, it may be possible to reveal, based on the inferred divergence dates between Kiwaidae and other chirostyloids, whether the age for this fossil is likely to be a stem-lineage kiwaid or chirostyloid. Three other fossils were identified as calibration points on the basis of being the earliest representative at a particular taxonomical level for that node.

(1) *Platykotta akaina* (Platykottidae) of Norian–Rhaetian age, 199.6–216.5 Ma. Earliest appearance of an anomuran in the fossil record [27].
(2) *Juracrista perculta* (Munididae) of Tithonian age, 145.5–150.8 Ma. Earliest appearance of Munididae in the fossil record [28].
(3) *Protaegla miniscula* (Aeglidae) of Albian age, 99.6–112 Ma. Earliest appearance of Aeglidae in the fossil record [29].

For details regarding the dating scheme and the dating priors in the Beast analyses, see the electronic supplementary material.

## 3. Results

### (a) Data summary and partitions

Of the 23 sequence sets produced, 16 were complete, five were missing a single gene fragment and two (*U. grandirostris* and *Calinectes sapidus*) were missing two gene fragments (see the electronic supplementary material, table S1). A total of 124 new DNA sequences were obtained and 95.7 per cent of the genes were successfully sequenced. Following PartitionFinder, the optimal partition scheme was a nine-partition dataset, with the three ribosomal genes treated separately and the six protein-coding genes split three ways into first, second and third codon positions.

### (b) Phylogenetic analyses

**Figure 3.**
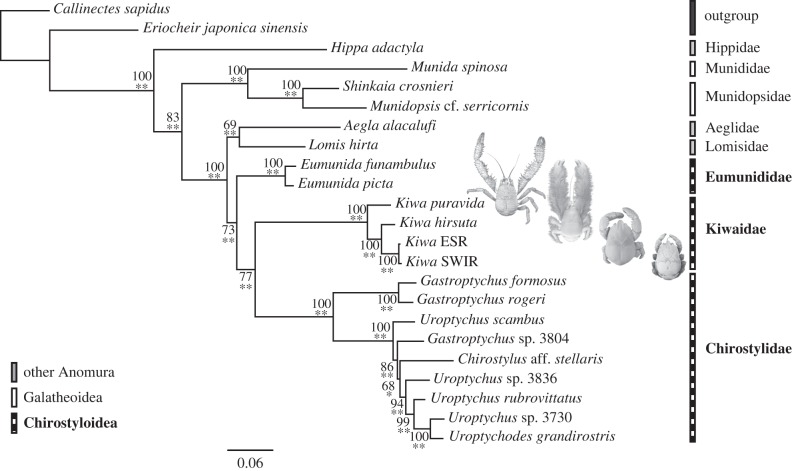
Maximum-likelihood (ML) and Bayesian topology of a nine-gene concatenated dataset with nine partitions. Node support numbers represent ML bootstrap percentages. Bayesian posterior probabilities are summarized as one asterisk for values more than 0.97 and two asterisks for values more than 0.99. Photographs of the four known kiwaids are superimposed next to their names. Photographs of *K. puravida* and *K. hirsuta* courtesy of Shane Ahyong from Thurber *et al*. [11] and Macpherson *et al*. [2], respectively.

Both the ML and BI analyses yielded identical tree topologies (figure 3). In general, node support was stronger in the BI analyses than in ML analyses, with posterior probabilities of greater than or equal to 0.97 for all nodes. In the ML analyses, 13 of the 20 nodes had bootstrap values greater than or equal to 99 per cent. The weakest bootstrap support was recorded for the clade comprising *Chirostylus* and four species of *Uroptychus* (68%). In general, weaker ML bootstrap support values compared with BI posterior probabilities are expected according to comparisons with simulated data [30].

The key features of the tree topology generated in this study are the monophyly of Aegloidea–Lomisoidea–Chirostyloidea, the monophyly of Chirostyloidea and, within it, the monophyly of Kiwaidae–Chirostylidae (figure 3). ML support for the Kiwaidae–Chirostylidae clade is not especially strong (77%), but the BI posterior probability was 1.00, and Bayesian topology hypotheses tests using the stepping-stone method supported this clade over Kiwaidae–Eumunididae (by 17.35 mean log likelihood units) and over Eumunididae–Chirostylidae (23.59 mean log likelihood units; electronic supplementary material, table S4).

Within Chirostylidae, the basal split is between *Gastroptychus* s.s, represented here by *G. formosus* and *G. rogeri*, and the remaining chirostylid taxa, including the second group of *Gastroptychus*, represented by *Gastroptychus* sp*.* 3804. *Gastroptychus*, as currently defined, is therefore not monophyletic. Likewise, the monophyly *Uroptychus* is not supported in this study. *Uroptychus scambus* resides outside a clade comprising the other *Uroptychus* species, *Chirostylus* aff. *stellaris, Gastroptychus* sp. 3804 and *U. grandirostris*. The location of *U. grandirostris* in the tree also renders the larger *Uroptychus* group paraphyletic (figure 3). All four species of *Kiwa* cluster together in this study, supporting the monophyly of Kiwaidae. There is a basal split between the seep-endemic *K. puravida* and a vent-endemic clade comprising *K. hirsuta* and the ESR and SWIR *Kiwa* species (figure 3).

### (c) Divergence time analyses

For ease of reporting, the median estimated divergence date is given, with the 95 per cent higher posterior density date range in parentheses. According to this study, Chirostyloidea split from sister taxa at 123.4 Ma (111.4–137.5 Ma). The divergences of the chirostyloid families occurred soon afterwards; Eumunididae split off at 114.8 Ma (101.3–129.5 Ma) and the split between Kiwaidae and Chirostylidae occurred at 106.4 Ma (92.8–121.1 Ma). Within Chirostylidae, the basal split between the *Gastroptychus* s.s clade and the other clades occurred at 73.5 Ma (61.2–87.2 Ma). The remaining clade radiated at 38.4 Ma (30.7–47.2 Ma). Extant Kiwaidae radiated at 30.6 Ma (22.7–39.3 Ma), with the split between the Pacific and non-Pacific lineages occurring at 19.1 Ma (13.4–25.9 Ma). The divergence between ESR and SWIR kiwaids was at 1.5 Ma (0.6–2.6 Ma).

## 4. Discussion

### (a) Phylogeny of Chirostyloidea

The higher-level phylogenetic patterns presented here are consistent with previous trees [5,6]. The monophyly of Aegloidea–Lomisoidea–Chirostyloidea supports the suggestion by Ahyong *et al*. [4] that, given the present-day locations of chirostyloids, aegloids and lomisoids (along with the fossil locations of aegloids and *Pristinaspina gelasina*), they all originated in the Pacific. Despite the shared characters between Eumunididae and Kiwaidae mentioned earlier, the monophyly of Kiwaidae–Chirostylidae is conceivable given their shared production of large eggs with highly abbreviated larval development, indicative of lecithotrophy [11,31]. In hydrothermal vent-endemic invertebrates, as well as in squat lobsters in general, mode of larval dispersal appears to be largely taxonomically constrained, rather than determined by habitat [32,33]. This accounts for the many dispersal strategies exhibited by vent-endemic fauna, despite being faced with the same challenges of dispersal from one ‘island’ to another [33]. Within Chirostylidae, the polyphyly of *Gastroptychus* and *Uroptychus* echoes the findings of Schnabel *et al*. [6], and this discrepancy between morphological taxonomy and molecular phylogenetics will have to be explored in more detail in the future.

The kiwaid phylogeny produced in this study has implications for our understanding of this family's evolutionary history, as well as the evolution of megafauna in chemosynthetic ecosystems in general. The Pacific location of the two basal kiwaids is consistent with a Pacific origin, as previously suggested [4], with a subsequent migration into the Atlantic sector of the Southern Ocean via the Drake Passage and then on to the Indian Ocean (figures 2 and 3). The alternative scenario—that Kiwaidae spread west from the Pacific into the Indian Ocean, and finally to Atlantic Sector of the Southern Ocean—seems unlikely as prevailing currents in the Southern Hemisphere are easterly and kiwaids are apparently absent further east in the Indian Ocean at the Central Indian Ridge. However, the basal split between a Northern Hemisphere kiwaid (*K. puravida*) and the Southern Hemisphere kiwaids, and the Alaskan location for the possible stem-lineage kiwaid fossil *Pristinaspina gelasina*, suggests a North Pacific origin for the family rather than the southern one previously proposed [4]. The tree topology revealed in this study also suggests that the body form with elongated chelae is most likely to be the ancestral state for extant kiwaids, with a trend of decreasing proportional chela length from Pacific species to the Southern and Indian Ocean species.

A noteworthy aspect of the kiwaid tree topology is the basal split between the cold seep lineage and the deeper vent lineages, consistent with the hypothesis that some fauna endemic to deep-sea hydrothermal vents evolved from ancestors that inhabited shallower, more temporally stable and less thermally extreme cold seeps on continental slopes [34]. Molecular phylogenetics shows some limited support for this hypothesis, at least with vestimentiferan tubeworms and mytilid mussels, where seep-endemic species generally fall out basally to the vent clades, as would be expected if vent fauna evolved from seep inhabitants [35,36]. The Pacific location for the seep-endemic *K. puravida* and the vent-endemic *K. hirsuta* suggests this seep-to-vent transition may have occurred along the eastern Pacific plate boundaries. The discovery of more extant kiwaid species, as well as fossils, may help to confirm this in the future. This seep-to-vent trajectory is part of a wider pattern seen in the fossil record whereby coastal lineages have subsequently radiated into offshore, deeper habitats, often with the eventual loss of their shallower relatives [37].

### (b) Coenozoic radiations in Chirostyloidea

**Figure 4.**
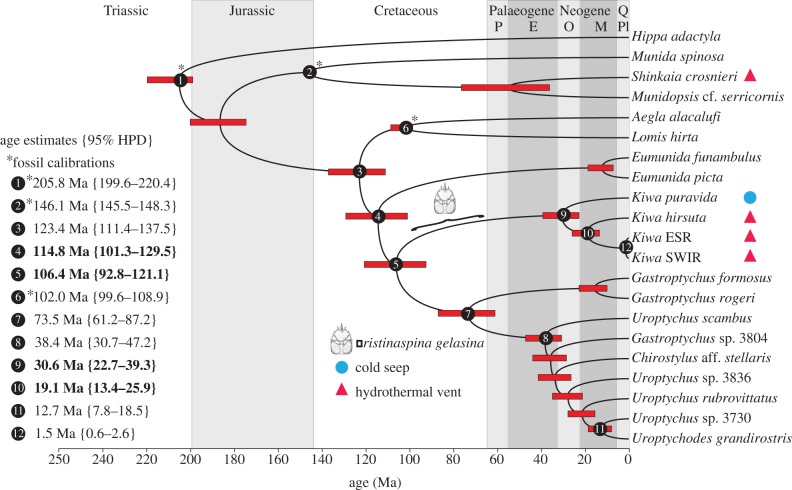
Divergence time estimates for the nine-gene concatenated dataset with nine partitions as calculated with a relaxed lognormal clock on Beast v. 1.7.4. Node bars represent the 95% highest posterior density (HPD) interval for nodal age. Numbered nodes show dates of interest to this study and quoted age values show median age estimates followed by the 95% HPD ranges in parentheses. Dates highlighted in bold are of particular interest. Nodes marked with an asterisk are fossil calibrated. Carapace illustration of the fossil *Pristinaspina gelasina* shows the date range for the fossil. Geological periods are shown at the top, with recent epochs represented as letters: P, Palaeocene; E, Eocene; O, Oligocene; M, Miocene; Pl, Plio-Pleistocene. Q, Quaternary. (Online version in colour.)

The Mid-Cretaceous origins (no later than 101.3 Ma) for the chirostyloid families (figure 4) indicate that *Pristinaspina gelasina* (65.5–99.6 Ma) cannot be a stem-lineage chirostyloid. These results are therefore consistent with the suggestion by Ahyong *et al*. [4] that this fossil is a stem-lineage kiwaid, based on its distinctive carapace markings (figure 1), although the possibility of it being a stem-lineage chirostylid–kiwaid cannot be completely ruled out as Kiwaidae and Chirostylidae diverged in 92.8–121.1 Ma. The dates for the formation of the three families are concomitant with a wider global pattern of decapod radiations that occurred during the Late Jurassic and Mid-to-Late Cretaceous, when eustatic sea levels were higher than they are today and there was an expansion of shallow, productive seas [38]. However, with the exception of the split between the *Gastroptychus* s.s clade and the remaining Chirostylidae, the radiations within Kiwaidae and Chirostylidae occur well into the Coenozoic, long after these two families diverged from one another. This pattern is consistent with limited fossil evidence suggesting the end of the Cretaceous was marked by the extinction of many decapod genera, but not families [39], which survived to the Coenozoic and subsequently re-radiated. The time frame for these radiations reported here coincides with a general intensification of global ocean circulation and possible deep-water ventilation from the Late Eocene/Oligocene onwards, following a warmer episode in the deep sea at the Palaeocene/Eocene boundary [40], perhaps allowing the exploitation of new niches in the deep sea.

The Coenozoic radiation of Kiwaidae augments the ever-expanding list of vent- and seep-endemic fauna that are now known to have recently evolved, rather than being considered ‘living fossils’ from the Mesozoic or Palaeozoic [41]. A comprehensive appraisal of the estimated radiation dates for vent and seep taxa suggests that most of them radiated after the Palaeocene/Eocene Thermal Maximum, a warm episode in the deep sea that may have resulted in widespread anoxia/dysoxia [42]. The results therefore reinforce the idea that chemosynthetic fauna may be vulnerable to reduction in oxygen levels in the deep sea as a result of changes to climate and ocean circulation, because they must occupy narrow redox zones at the limit of their physiological tolerance [42]. The fact that Kiwaidae radiated (or re-radiated) recently is reflected by their association with ectosymbiont bacteria, which, in terms of host–symbiont relationships, may be an early evolutionary step towards more intimate symbiotic associations with bacteria [43] (e.g. the housing of chemosynthetic symbionts in specialized internal organs [13]). It is notable that other decapods associated with ectosymbionts, the galatheoid squat lobster genus *Shinkaia* and the shrimp family Bresiliidae, may also have Coenozoic origins, based on fossil and molecular evidence respectively [44,45].

### (c) Vicariance in vent-endemic Kiwaidae

Vent-endemic fauna maintain populations along ridges by broadcasting their larvae from vent field to vent field. Species ranges are determined by factors such as larval longevity, current direction and strength distance between vent fields, shelf and ridge topography, and vent field longevity [46]. In general, vent community similarity is determined by along-ridge axis distance between vents rather than the shortest distance along the seafloor [47], because bottom currents are often rectified by ridge topography, thus entraining larvae along ridge axes [46]. In some cases, consequently, the biogeography of hydrothermal vent-endemic fauna can be understood in terms of vicariance caused by past changes in mid-ocean ridge position [48]. Such events may also be responsible for the divergence of vent-endemic Kiwaidae, but explaining present-day biogeographic patterns can be problematic, as tectonic and oceanographic reconstructions become more uncertain with distance into the past.

A key question in the biogeography of Kiwaidae is how they managed to spread from vents in the Pacific to those on the ESR and SWIR. The known present-day locations of Kiwaidae (figure 2) in combination with the phylogeny present here suggest that they entered the Atlantic sector of the Southern Ocean from the Pacific via the Drake Passage. The estimated date range for the split between the Pacific and non-Pacific lineages (13.4–25.9 Ma) is compatible with this scenario, as the deep-water connection in the Drake Passage probably occurred around 33 Ma [49].

**Figure 5.**
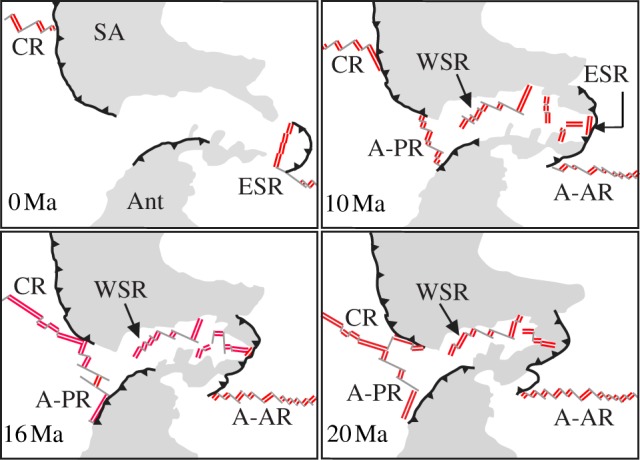
Diagram representing the evolution of ridge positions in the Drake Passage relevant to the divergence of Pacific and non-Pacific kiwaids during the Oligocene and Miocene, modified from Vérard *et al*. [49] and Breitsprecher & Thorkelson [50]. Grey areas represent non-oceanic plate regions. Double lines denote active spreading segments of the ridges. Grey lines represent faults and fracture zones. Solid black lines with triangles denote subduction zones. SA, South America; Ant, Antarctica; CR, Chile Rise; ESR, East Scotia Ridge; A-PR, Antarctic–Phoenix Ridge; WSR, West Scotia Ridge; A-AR, American–Antarctic Ridge. (Online version in colour.)

Today, the ESR is isolated from the Pacific ridge systems and the means by which kiwaids arrived from the Pacific into the Scotia Sea is not readily apparent. However, at approximately 20 Ma, there was a nearly continuous chain of ridge segments from the Pacific into the widening Scotia Sea via the Chile Rise, Antarctic–Phoenix Ridge and the West Scotia Ridge (WSR) [49] (figure 5*d*). The ESR was forming by approximately 15 Ma [51] at the eastern end of the WSR and by 12 Ma the subducting Chile Rise had left a gap of approximately1000 km between the Pacific Ridges and the WSR–ESR system [49,50] (figure 5*b–d*). This subduction under the South American plate, starting at approximately 16 Ma, coincides with the most recent divergence date estimate for the Pacific and non-Pacific kiwaids (13.4 Ma). This event is not the only candidate, however. On the Chile Rise at approximately 28–26 Ma, there was a nearly 90° realignment in the axis of spreading on the Chile Rise, resulting in the formation and subsequent expansion of large fracture zones [52], which could have isolated vent fauna on the Pacific–Antarctic Ridge from Chile Rise populations. The oldest possible inferred divergence date of 25.9 Ma (figure 4) is close enough to this event for it to be worth considering as a cause of the divergence we see today. Discovering kiwaids on the as-yet-unexplored Chile Rise may resolve this question.

The divergence between the ESR and SWIR kiwaids is very recent compared with the other kiwaids (as recently as 0.6 Ma). During this time, there have been no major changes in ridge configuration between the ESR and SWIR to easily account for such a divergence [53,54]. One possibility is that there has been a recent drop in the number of hydrothermal vent fields along portions of the intervening ridges, which would have reduced the dispersal capability of vent fauna by effectively increasing the distance between adjacent vent fields, leading to isolation and subsequent divergence. Alternatively, changes in current regime may be responsible; large portions of the intervening ridge segments between the ESR and the SWIR are bathed by the Antarctic Circumpolar Current (ACC), which is the dominant force in determining the dispersal direction of larvae throughout the Southern Ocean [55]. Changes to the ACC could have affected the dispersal range of *Kiwa* larvae, and in particular their ability to traverse large potential barriers to gene flow, such as the Andrew Bain Fracture Zone (ABFZ) [56], which effectively splits the SWIR into a lower and an upper portion (figure 2).

Today, the Subantarctic Front and Polar Front of the ACC cut across the ABFZ [57], potentially isolating vent fauna on either side. Changes in the intensity and latitude of the ACC fronts during the Mid-Pleistocene Transition, which occurred between approximately 1.2 Ma and 650 ka, could have transported *Kiwa* larvae across the ABFZ to regions that are now isolated. During this episode, orbitally forced glacial cycles switched in periodicity from 41 to 100 kyr cycles, resulting in colder, extended glacial conditions and northerly shifts in the ACC polar front in the South Atlantic far beyond the northerly extent of recent glacial front migrations [58]. Sediment analyses off the Antarctic Peninsula indicate that there has been a decline in ACC strength since approximately 2.5 Ma [59], which might have cut off the supply of *Kiwa* larvae across fracture zones such as the ABFZ at some point. Exploration of the American–Antarctic Ridge and lower reaches of the SWIR around the Bouvet Triple Junction may elucidate present-day barriers to gene flow between the ESR and SWIR kiwaids, and help in the inference of past changes responsible for their divergence. The investigation of vent fields east of Dragon will aid in determining the extent of this genus on the SWIR, but at a wider scale the discovery of vent communities along the Southeast Indian Ridge and along the Pacific–Antarctic Ridge will help reveal the global extent of vent-endemic Kiwaidae.

## 5. Conclusion

The nine-gene dataset featured in this study has revealed, in accordance with previous work, that Chirostyloidea are monophyletic. However, in contrast to earlier studies, our results suggest the monophyly of Kiwaidae–Chirostylidae, which is supported morphologically by their similar larvae. Within Chirostylidae, *Uroptychus* and *Gastroptychus* are polyphyletic and need taxonomic re-examination. All three families appear to have Mid-Cretaceous origins, although kiwaids and some chirostylids radiated after the Late Eocene. The basal split in Kiwaidae between the seep-endemic *K. puravida* and a vent-endemic clade is consistent with the seep-to-vent hypothesis, although more evidence is needed to determine this. The vent clade then probably spread via mid-ocean ridges from the East Pacific, through the Drake Passage to the ESR and SWIR within the last 25.9 million years. Similar to many other chemosynthetic taxa, the Coenozoic radiation of Kiwaidae may indicate an inherent vulnerability of chemosynthetic fauna to climatic changes affecting the availability of oxygen in the deep sea, with consequences for their future conservation.
